# Smart needle electrical bioimpedance to provide information on needle tip relationship to target nerve prior to local anesthetic deposition in peripheral nerve block (USgPNB) procedures

**DOI:** 10.2478/joeb-2023-0007

**Published:** 2023-12-31

**Authors:** Edel Whelton, Lisa Helen, Brian O’Donnell, Martin O’Sullivan, Justina Ugwah, Walter Messina, Yineng Wang, Niamh O’Mahoney, Eric Moore

**Affiliations:** 1 Life Science Interface, Tyndall National Institute, University College, Cork, Ireland; 2 Cork University Hospital, Cork, Ireland; 3 School of Chemistry, University College Cork, Ireland

**Keywords:** Bioimpedance, current, voltage, peripheral nerves, connective tissue, neural tissue, anesthetic, smart needle

## Abstract

Ultrasound guided peripheral nerve block (USgPNB) refers to anaesthetic techniques to deposit local anesthetic next to nerves, permitting painful surgery without necessitating general anesthesia. Needle tip position prior to local anesthetic deposition is a key determinant of block success and safety. Nerve puncture and intra-neural injection of local anesthetic can cause permanent nerve injury. Currently ultrasound guidance is not sufficiently sensitive to reliably detect needle to nerve proximity. Feedback with bioimpedance data from the smart needle tip might provide the anesthetist with information as to the relationship between the needle tip and the target nerve prior to local anesthetic deposition.

Bioimpedance using a smart needle integrated with a two-electrode impedance sensor has been developed to determine needle to nerve proximity during USgPNB. Having obtained all necessary ethical and regulatory approvals, *in vivo* data on brachial plexus, vagus, femoral and sciatic nerves were obtained from seven pig models using the smart needle bioimpedance system. The excision and histological analysis of above peripheral nerves and observation of the architecture and structure of nerves by means of histology allow the calculation of the ratios of connective tissue to neural tissue to determine the influence of this variable on absolute impedance. The ratio results give extra clinical data and explain the hetrogeneity of impedance data in the pig models and the hypothesis that connective tissue with intra-neural fat has higher impedance than neural tissue.

## Introduction

This research was undertaken with the aim of developing a novel fabrication process to enable integration of a two-electrode system to a hypodermic needle and therefore fabrication of a smart needle. The concept was conceived with the intention of addressing a currently unmet clinical need for a regional anesthetic procedure, ultrasound guided peripheral nerve block (USgPNB). The technique USgPNB refers to a set of medical procedures which block nerve impulse conduction, provide anesthesia and pain relief to facilitate surgical operations or are performed to treat acute or chronic pain. The location of the needle tip relative to the target nerve is crucial to the safe and effective practice of USgPNB. By developing a smart needle, a hypodermic needle integrated with an impedance sensor, bioimpedance can be measured at the needle tip.

Bioimpedance has been used to differentiate between tissue types and therefore has the potential to identify the tissue type located at the needle tip, identifying the needle location within the body. Accurate identification of tissue at the needle tip could provide valuable objective information and inform high stakes procedural decisions prior to injection of local anesthetic in close proximity to neural structures. The inability to objectively identify the exact needle position in relation to nerve structures during USgPNB has been identified as a limitation of the technique [1,2]. Ultrasound guidance has brought significant advancement in the performance of PNB which began by estimating nerve location using anatomical landmarks and knowledge and progressed in later years by the introduction of the nerve stimulation technique.

Now ultrasound guidance enables direct visualisation of target nerves, the needle and spread of anesthetic once the drug has been injected. Despite the fantastic advances ultrasound imaging has provided to the PNB procedure, the subjectivity of interpreting ultrasound images and the inability to objectively identify needle to nerve contact or intra-neural needle positioning has led to the investigation of ways to enhance the technique further by providing information on the identity of tissue type at the needle tip. “Regional anaesthesia always works—provided you put the right dose of the right drug in the right place” [12].

Perioperative nerve injury may occur following anaesthesia and surgery [2,14]. The true incidence of nerve injury following all forms of anaesthesia is unknown. Most data are derived from analyses of closed claims databases in the USA, whereby there is a surprising association between nerve injury and general anaesthesia. The best contemporaneous estimates of nerve injury frequency following peripheral nerve block suggest that injury occurs in 4-6 per 10,000 nerve blocks performed [7,13]. Although rare, iatrogenic nerve injury can have severe implications for the person affected. It may result in permanent loss of sensation and power affecting the area supplied by the injured nerve. Chronic neuropathic pain may accompany the condition. The resultant loss of limb function and chronic pain can have catastrophic physical, psychological, social and economic consequences for the injured party [15].

**Fig.1:**
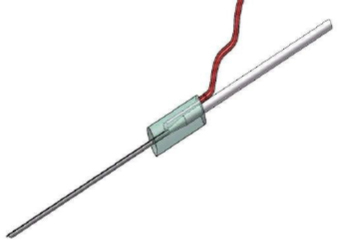
Concept drawing of the smart needle with drug delivery tube and electrical connections.

### 1.1 Electrical Impedance (EI) to distinguish intra-neural from extra-neural needle placement

USgPNB is one example of a procedure that could benefit from a smart needle, a hypodermic needle integrated with an impedance sensor, to determine needle tip location within the body. Limitations remain with the inability to objectively identify the relationship of the needle tip to the nerve prior to injection during USgPNB as mentioned previously. To address this limitation, a hypodermic needle integrated with an impedance sensor was developed that could potentially identify tissue at the needle tip thereby providing objective information indicative of needle tip location. Electrical impedance was identified as a feasible parameter, as impedance has previously been used successfully to detect tissue types in diverse clinical scenarios. The possibility of distinguishing intra-neural from extra-neural tissue based on bioimpedance has already been demonstrated and Kalvøy’s group have made novel investigations regarding needle guidance using bioimpedance [3]. However, additional data is needed to confirm the sensitivity and reproducibility of the technology.

### 1.2 Ratios & microstructures

The ratio of neural tissue to non-neural tissue between the different nerves and between different needle positions in the same nerve could be a substantial source of variation in bioimpedance trends for *at-nerve* and *in-nerve* test sites. Moayeri et al. quantified the neural and non-neural components of the brachial plexus and the sciatic nerve and investigated how the ratio of neural to non-neural tissue changed from proximal to distal. As the nerve leaves the spinal cord, the density of an epineurium (composed of stroma and connective tissue) decreases, but the total volume increases. Thus, the ratio of neural to non-neural tissue is approximately 1:1 in proximal plexuses, 1:2 in distal plexus and a peripheral nerve might contain up to 70% of connective tissue [5, 6].

This means more stroma and connective tissue is present within the epineurium of the brachial plexus the further away the nerve travels from the spinal cord. As the vagus nerve was used to obtain bioimpedance data from in this animal study, the proximal ratio of neural to non-neural tissue is more relevant for this data set as the vagus nerve lies proximal to the brachial plexus.

The sciatic nerve is similar to other peripheral nerves with outer epineural tissue surrounding fat and connective tissue and internal nerve fascicles surrounded by perineurium. The sciatic nerve is divided into tibial and common peroneal compartments in humans. Each compartment is composed of fascicles enveloped by their own epineural tissue. The two compartments, within the sciatic nerve, are separated by a connective tissue-adipose septum, known as the Compton Cruveilhier septum, which runs internally, along the length of the sciatic nerve [4]. The sciatic nerve is flat and large in shape proximally (closer to the spinal cord) and becomes more oval and smaller distally (as it moves further away from the spinal cord). This is related to the size and architecture of the brachial plexus, which changes from an oligofascicular (few fascicles) to a multifascicular pattern as it travels distally [4]. The sciatic nerve is multifascicular throughout and has a different microanatomy. The absolute amount of neural tissue decreased from proximal to distal whereas the non-neural tissue remained about the same. This decrease is attributed to branching off nerve tissue to the upper leg muscles. Consequently, neural to non-neural tissue ratio changed, from proximal to distal, from 2:1 to 1:1 [7, 2].

## Materials and methods

### 2.1 Electrical impedance (EI) measurement in a freshly slaughtered pig carcass

The first indication of nerve bioimpedance was obtained during the pig carcass study performed at a local abattoir. Nerve bioimpedance could not be obtained during *in vitro* studies as nerve tissues disintegrate and are absorbed by surrounding tissues within approximately 2 hours of death of the animal. Therefore, nerve tissue is not present in meat. For this reason, it was necessary to travel to a local abattoir and gain access to a pig carcass as soon as possible post-slaughter. The animal used for the pilot study was made available to us within 10 minutes of slaughter. All standard operating procedures for slaughter of the animal was adhered to and remain unaffected.

### 2.2 Electrical impedance measurement of tissues in an anesthesised pig model

Prior to device validation in an anesthetised animal model, *in vitro* bioimpedance measurements were performed on pork meat samples. Results demonstrated a clear distinction between muscle and subcutaneous tissues based on bioimpedance recordings. In pigs, femoral nerve, sciatic nerve, and brachial plexus (vagus nerve) sites were used to obtain bioimpedance recordings from subcutaneous tissue, muscle and *at-nerve* and *in-nerve* tissues. Data was obtained from 7 animals initially using the frequency range of 10 – 100 kHz, voltage at 10 mV, current range 100 nA – 1 mA on the first two animals and a change in frequency range to 100 Hz – 1 MHz for the other five animals. The frequency range for bioimpedance measurement was widened to ensure as much useful data as possible was collected thus increasing the probability of identifying unique bioimpedance trends for tissues at certain frequencies within the range. If necessary, the optimum frequency range could be narrowed again after identification and characterisation of unique tissue bioimpedance profiles with the wider range.

**Fig.2:**
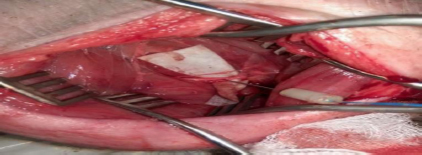
Smart needle positioned in the sciatic nerve [11].

**Fig.3:**
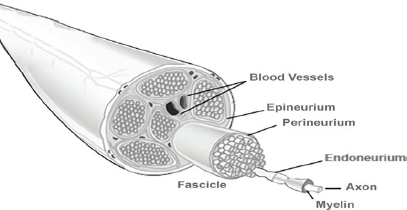
Schematic of possible needle position within a nerve structure for bioimpedance measurement [11].

The vagus nerve (a nerve lying closer to the spinal cord than the brachial plexus) at the side of the neck was easier to access than the brachial plexus. For this reason, the side of the neck was open dissected to the vagus nerve instead of exposing the brachial plexus. Therefore, bioimpedance data was obtained from the different tissues around the vagus nerve on day 4 – 7 of the animal study.

### 2.3 Haemogylin Van Goss stained slides of excised femoral, sciatic & vagus peripheral nerves and ratio calculations of connective to neural tissue using ImageJ software

Femoral, sciatic, brachial and vagus nerves were excised from the nerve locations where the bioimpedance measurements were analysed to validate the nerve type. These slides were prepared in histopathology at Cork University Hospital (CUH) with Haemogylin Van Goss Stain on cross section and longtidutional peripheral nerves. These stained peripheral nerve slides were observed under an electron microscope in life sciences laboratory at Tyndall National Institute (TNI). ImageJ software was used, and clear images of peripheral nerves were exported and analysed. Sketching around the parameters of connective and neural tissue separately the ratios of connective to neural tissue in each peripheral nerve could be calculated.

#### Ethical approval

The research related to animal’s use has been complied to, with all the relevant national regulations and institutional policies for the care and use of animals.

## Results

### 3.1 Electrical impedance measurement in a freshly slaughtered pig carcass

Bioimpedance results from the ultrasound guided tissue identification procedure in the pig carcass did not match previous *in vitro* experimental results. In particular muscle bioimpedance under ultrasound guidance was uncharacteristically high. The tissues during this procedure were identified from the ultrasound image by a consultant anesthetist, an expert in interpretation of ultrasound images of human anatomy.

**Fig.4:**
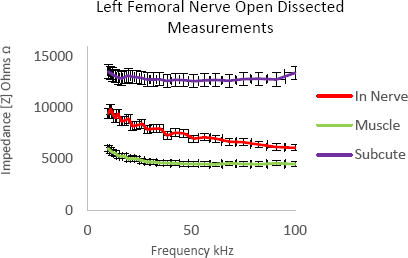
Bioimpedance of fat, muscle, and nerve in a freshly slaughtered pig carcass.

This was the first opportunity for the expert in human anatomy to ultrasound image porcine anatomy and attempt to locate and identify different tissue structures. The bioimpedance results on open dissection of the tissues fig. 4 were much closer to what was expected from previous *in vitro* results where muscle had low bioimpedance.

This was the first and only attempt to date at obtaining bioimpedance of porcine tissues in a freshly slaughtered animal. During bioimpedance measurement of nerve tissue it was noted how fragile and friable the nerve structure was. The nerve was already beginning to disintegrate within the surrounding tissues as measurement was performed thus the bioimpedance results obtained should only represent a rough estimate of the bioimpedance of nerve in live tissue. This pilot study however was not only allowed an indication of bioimpedance of near to live porcine tissues but also allowed testing of a study protocol before use on a live anaesthetized pig. Insights gained from this pilot study enabled refinement of the protocol for bioimpedance measurement of *in vivo* tissues and was an important step in progressing to a live anaesthetized pig model [11]. The decision to continue using open dissection method on the *in vivo* pig models was to ensure accuracy of data for this preliminary study as the needle prototype is small in length.

### 3.2 Electrical impedance measurement of tissues in an an-esthetised pig model femoral, sciatic & vagus nerves sites

On observation of the tissue bioimpedance data from live tissues in the first two animals evaluated, it was found to be necessary to assess the repeatability and reproducibility of data provided by the smart needle in live tissues.

Results from these studies, along with pre- and post-testing impedance measurement of NaCl solutions, demonstrated that the data obtained using the smart needle was repeatable and had good reproducibility for most tissues. However, some variation between puncture sites was observed during reproducibility testing [11]. This was expected due to the heterogeneity of biological tissues and was accounted for by taking three bioimpedance measurements at three different puncture sites within proximity to each other, from each tissue at each test site [2].

With respect to this research and the intended application of the technology described, the bioimpedance profiles of the *in-nerve* and *at-nerve* locations were of most significant interest. It was found that at times no differentiation between these two locations was provided by bioimpedance data. When differentiation between these locations was observed the femoral and vague nerve sites displayed higher EI for *at-nerve* than *in-nerve* while the opposite was found for the sciatic nerve site (*at-nerve* had lower EI than *in-nerve*). It must be noted there were exceptions to this trend at all sites also [11]. Interestingly, despite the sciatic nerve data obtained indicating the *at-nerve* site had lower bioimpedance than *in-nerve*, which was the inverse of what was found at the femoral and brachial nerve sites. This data correlates with the findings of a previously published study on distinguishing intra-neural from extra-neural needle placement using a nerve stimulator to measure impedance [1, 2].

### 3.3 Haemoxylin Van Goss Stained Slides of Excised Femoral, Sciatic & Brachial Peripheral Nerves and Ratio Calculations of Connective to Neural Tissue using Image J Software

## Discussion

**Fig.5:**
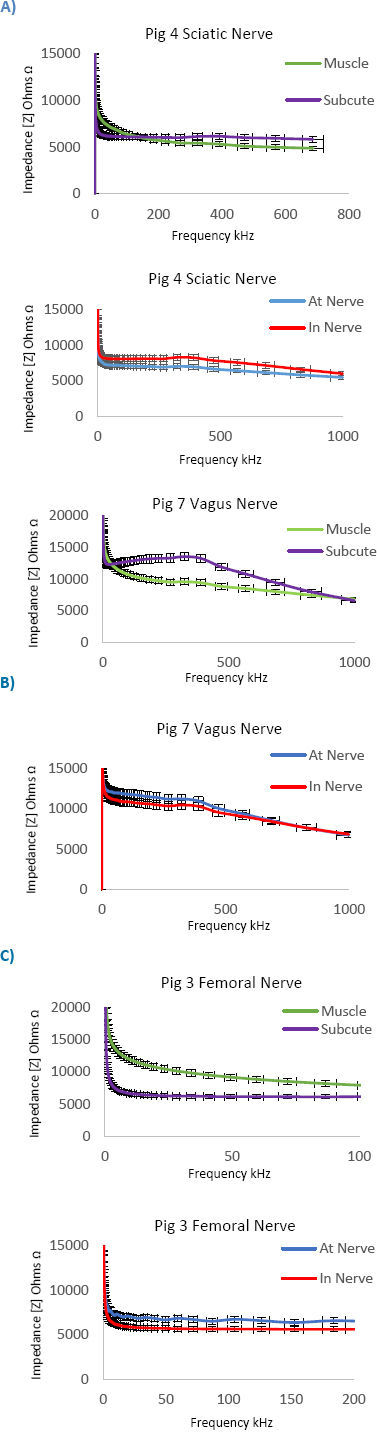
Bioimpedance results of Sciatic A), Vagus B) and Femoral C) peripheral nerves demonstrating higher impedance at-nerve for femoral and brachial nerve and lower impedance in-nerve. The sciatic nerve has lower impedance at-nerve and higher impedance in-nerve.

**Fig.6:**
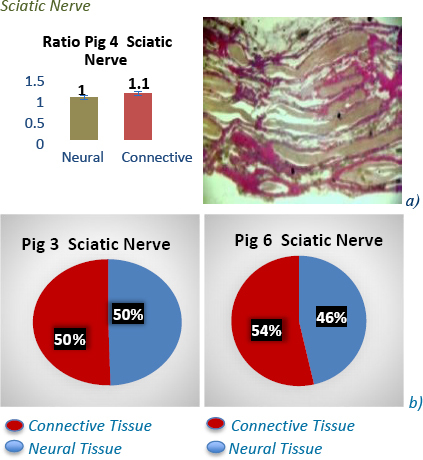
Ratio of neural to connective tissue in sciatic nerve in pig model 4 is represented in a) above & image of a stained sciatic nerve clearly defining locations of neural and connective tissue. Percentages of neural to connective tissue in pig 3 & 6 are represented in b) above demonstrating Percentages in other pig models are close to ratio of 1:1 in sciatic nerves evaluated.

**Fig.7:**
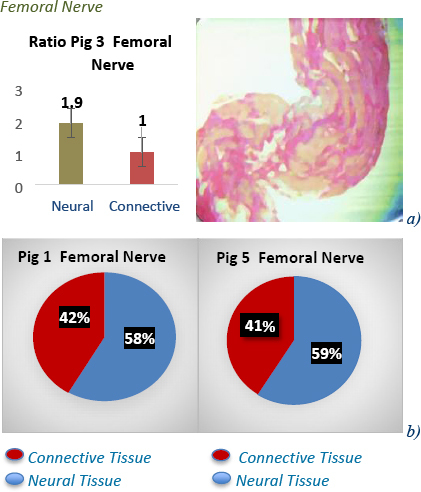
The ratio of neural to connective tissue in femoral nerve in pig model 3 is represented in a) above & image of a stained femoral nerve clearly defining locations of neural and connective tissue. The percentages of neural to connective tissue in pig 1 & 5 is represented in b) above demonstrating Percentages in other pig models are close to ratio of 1.9:1 in femoral nerves evaluated.

**Fig.8:**
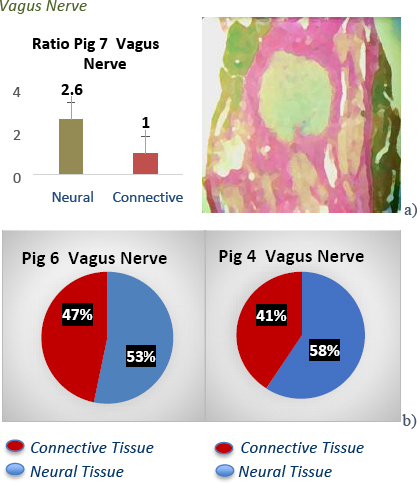
Ratio of neural to connective tissue in vagus nerve in pig model 7 is represented in a) above & image of a stained vagus nerve clearly defining locations of neural and connective tissue. Percentages of neural to connective tissue in pig 6 & 4 are represented in b) it can be observed that neural tissue is more abundant than connective tissue in other pig models in vagus nerves evaluated when compared to pig model 7.

The histological samples taken at test sites were used to confirm tissue type present at that site and further studied with the ImageJ software and ratios of connective and neural tissue calculated. This study showed a lower impedance observed in the sciatic *at-nerve*, and higher impedance *in-nerve* with ratios 1:1 neural to connective tissue across most models Figures 5 A) & 6. In the brachial plexus, vagus and femoral nerves, a higher impedance was observed *at-nerve* and lower impedance *in-nerve*, and the ratios have more neural tissue to connective tissue across most models figures 5 B) & C) and figures 7 & 8. These results give extra clinical data and explain the heterogeneity of impedance data in the pig models and the hypothesis that connective tissue with intra-neural fat perhaps has higher impedance than neural tissue. These neural to non-neural tissue ratios and how they change depending on location along the nerve demonstrate how different architecturally peripheral nerves are from each other, as well as having different microanatomies.

These characteristics would influence the bioimpedance of tissues at separate locations and, therefore must be considered when interpreting data and planning any future validation experiments. The results presented within this document, along with evidence of different microanatomies within different peripheral nerves, indicate that the EI properties at each major peripheral nerve site need to be characterised individually. The true location of the sensor on the smart needle once the needle was inserted into the nerve was unknown. Variability of results may also have been influenced by the section of nerve exposed and the location of measurement Figure 2. Clinically, even if a needle enters a peripheral nerve, it might still be inside the connective tissue, not necessarily within the neural fascicle. This also explains the longer block onset times for peripheral nerve blocks as opposed to proximal plexus blocks. Again a few scenarios that could have been possible include the sensor being in contact with (A) neural tissue, (B) non- neural tissue i.e., stroma and connective tissue or (C) may have passed through to the other side of the nerve structure to the outer layer of nerve tissue at the opposite side of the nerve. Again, any or all these scenarios could have affected the data recorded in any of the tests.

## Conclusion

It is not possible to determine with accuracy if the needle tip is placed *at-nerve, in-nerve* (connective tissue, stroma, or neural tissue). The understanding of microstructure and ratios of connective to neural tissue in peripheral nerves and their impedance values will allow for greater specificity of needle tip placement. The only nerve site investigated by our study that can be compared with results obtained by Tsui et al. and Vydyanathan et al. was nerve tissue at the sciatic nerve site. However, parameters used by Tsui et al. and Vydyanathan et al. differ from this study; 1 Hz, 0.1 ms and 0.5 mA and 2 Hz, 0.1 ms, 0.5 mA, respectively. The main findings remain as intra-neural (*in-nerve*) bioimpedance is higher than extra-neural (*at-nerve*) bioimpedance at the sciatic nerve. The inverse bioimpedance trend for *in-nerve* and *at-nerve* locations at the sciatic nerve site compared to the femoral and vagus nerve site could be due to the difference in the nerve’s microanatomy [1].

Further data collection on the real-time bioimpedance measurement of peripheral nerves with a commercially produced Smart Needle and the development of an in-clinical trial would support the animal study. Bioimpedance study of specific needle tip insertion into connective and neural tissue separately, with observation of ratios obtained through histological analysis is required to validate the above hypothesis.

Going forward a data bank of impedance readings needs to be compiled of *at-nerve* sites of each specific peripheral nerve and *in-nerve* neural tissue, connective tissue, and stroma; enabling a trend to be observed, therefore reliability of needle placement aiding in avoiding nerve injury.

## References

[1] Tsui B.C. (2008). Electrical Impedance to Distinguish Intraneural from Extraneural Needle Placement in Porcine During Direct Exposure and Ultrasound Guidance. Anaesthesiology.

[2] Vydyanathan A. (2016). The Use of Electrical Impedance to Identify Intraneural Needle Placement in Human Peripheral Nerves: A Study on Amputated Human Limbs. Anesth Analg.

[3] Kalvøy H, Sauter A.R. (2016). Detection of Intraneural Needle Placement with Multiple Frequency Bioimpedance Monitoring: A Novel Method. Journal of Clinical Monitoring and Computing.

[4] Moayeri N. (2010). Correlation among Ultrasound, Crosssectional Anatomy, and Histology of the Sciatic Nerve: A Review. Reg Anesth Pain Med.

[5] Van Geffen G.J. (2009). Correlation between Ultrasound Imaging, Cross-Sectional Anatomy, and Histology of the Brachial Plexus: A Review. Reg Anesth Pain Med.

[6] Moayeri N, Bigeleisen PE, Groen GJ. (2008). Quantitative architecture of the brachial plexus and surrounding compartments, and their possible significance for plexus blocks. Anesthesiology.

[7] Moayeri N., Groen G.J. (2009). Differences in Quantitative Architecture of Sciatic Nerve May Explain Differences in Potential Vulnerability to Nerve Injury, Onset Time, and Minimum Effective Anesthetic Volume. Anesthesiology.

[8] Helen L, O’Donnell B, Messina W, O’Mahony C, Ahmed OMA, Moore EJ. (2017). Impedance Sensor to Detect Substance Change at the Needle Tip. Electroanalysis.

[9] Helen L, O’Donnell BD, Moore E. (2015). Nerve localization techniques for peripheral nerve block and potential future directions. Acta Anaesthesiol Scand.

[10] Helen L, Messina W, O’Donnell B, Moore E. (2014). Investigation of tissue bioimpedance using a macro-needle with a potential application in determination of needle-to-nerve proximity. Liverpool: International Journal on Smart Sensing and Intelligent Systems.

[11] Helen L. (2018). Development of a smart needle integrated with an impedance sensor to determine needle to nerve proximity for nerve blocking(anaesthetic) procedures.

[12] Denny N.M., Harrop-Griffiths W. (2005). Location, Location, Location! Ultrasound Imaging in Regional Anaesthesia. British Journal of Anaesthesia.

[13] Cheney F.W. (1999). Nerve Injury Associated with AnesthesiaaClosedClaimsAnalysis. Anesthesiology.

[14] Kroll D.A. (1990). Nerve Injury Associated with Anesthesia. Anesthesiology.

[15] Auroy Y. (2002). Major Complications of Regional Anesthesia in France: The Sos Regional Anesthesia Hotline Service. Anesthesiology.

